# PathFX provides mechanistic insights into drug efficacy and safety for regulatory review and therapeutic development

**DOI:** 10.1371/journal.pcbi.1006614

**Published:** 2018-12-07

**Authors:** Jennifer L. Wilson, Rebecca Racz, Tianyun Liu, Oluseyi Adeniyi, Jielin Sun, Anuradha Ramamoorthy, Michael Pacanowski, Russ Altman

**Affiliations:** 1 Department of Bioengineering, Stanford University, Palo Alto California, United States of America; 2 Division of Applied Regulatory Science, US Food and Drug Administration, Silver Spring Maryland, United States of America; 3 Office of Clinical Pharmacology, Office of Translational Sciences, Center for Drug Evaluation and Research, US Food and Drug Administration, Silver Spring Maryland, United States of America; 4 Department of Genetics, Stanford University, Palo Alto California, United States of America; Icahn School of Medicine at Mount Sinai, UNITED STATES

## Abstract

Failure to demonstrate efficacy and safety issues are important reasons that drugs do not reach the market. An incomplete understanding of how drugs exert their effects hinders regulatory and pharmaceutical industry projections of a drug’s benefits and risks. Signaling pathways mediate drug response and while many signaling molecules have been characterized for their contribution to disease or their role in drug side effects, our knowledge of these pathways is incomplete. To better understand all signaling molecules involved in drug response and the phenotype associations of these molecules, we created a novel method, PathFX, a non-commercial entity, to identify these pathways and drug-related phenotypes. We benchmarked PathFX by identifying drugs’ marketed disease indications and reported a sensitivity of 41%, a 2.7-fold improvement over similar approaches. We then used PathFX to strengthen signals for drug-adverse event pairs occurring in the FDA Adverse Event Reporting System (FAERS) and also identified opportunities for drug repurposing for new diseases based on interaction paths that associated a marketed drug to that disease. By discovering molecular interaction pathways, PathFX improved our understanding of drug associations to safety and efficacy phenotypes. The algorithm may provide a new means to improve regulatory and therapeutic development decisions.

## Author summary

Many drugs fail to reach the market because they are not sufficiently efficacious for their disease indication or they cause intolerable side-effects. To understand drug efficacy and safety, we created an algorithm, PathFX. The algorithm identified relationships between drugs and diseases, and drugs and side-effects. We tested PathFX’s ability to identify the disease for which the drug was developed. We applied PathFX to post-marketing reports of drug side effects and identified drug side effects where regulatory review was ambiguous. Finally, we identified novel diseases for which marketed drugs could treat. The method has the potential to be a tool for assessing drug safety and efficacy during development and may have utility for regulators and industry scientists.

## Introduction

The drug discovery process is long, difficult, and expensive. Only ~10% of drugs entered in human studies make it to the market[1] because many drugs have insufficient efficacy[2] or significant safety issues[3,4]. The lack of efficacy may be related to poor bioavailability, incomplete inhibition of the target, or selection of a target that is not a central driver of the disease. Adverse events can occur through primary effects of a drug on the intended targets or respective biological pathways, or secondary effects that can occur with off-target binding [3,5]. For these reasons, understanding a drug’s phenome–the collection of clinical characteristics that are related to a drug target or pathway–is integral to validating and prioritizing drug targets for development, and identifying other potential (adverse) drug effects that may occur by perturbing a particular biological network. However, the available tools for characterizing drug pathways during target optimization and regulatory review catalogue varied aspects of the cellular response to drug and integration across these resources is often incomplete.

Many data sources containing relevant information are available to characterize pharmacological drug effects. However, the vast amount of information is siloed into separate databases, creating a patchwork of information that is difficult and tedious to easily integrate for regulatory review. To illustrate, in evaluating a drug that inhibits a specific enzyme, one might look to Mendelian disease as an indicator of what clinical manifestations are related to loss of that enzyme’s function; this could inform what toxicities or benefits might arise from pharmacologically interrupting the enzyme’s function (e.g., as for the case of PCSK9). This exercise can then be repeated by evaluating the genetic epidemiology of more common variations in the enzyme, and so on. Relevant resources that could collectively inform a drug target’s phenome include Pharmacogenomics Knowledgebase (PharmGKB)[6], genome-wide association studies (GWAS)[7], Online Mendelian Inheritance in Man (OMIM)[8], disease-gene associations such as DisGeNet[9,10], and phenotype-gene association studies (PheWAS)[11,12]. In addition, focusing on a single gene or protein does not always provide a complete view of the biological milieu, or full pathway context, relevant to a drug target. Protein interaction databases, such as STRING[13,14] and iRefWeb[15], relate drug targets to signaling intermediates to provide context for single gene effects, though, these are not easily linked to phenotype information.

Network methods can be useful for identifying mechanistic interactions that relate drug targets to adverse reactions, and are under-utilized in understanding drug adverse effects[5]. A network approach uncovered interaction intermediates between drug targets associated with peripheral neuropathy[16], drug-induced rhabdomyolysis[17], drug-induced severe cutaneous stevens-johnson syndrome[18], drug-induced lung injury[19], and drug-induced contraction-related cardiotoxicity[20]. A further meta-analysis of networks for these toxicities discovered protein mediators that are common among pairs of toxicities[20]; for instance, they discovered that drugs associated with peripheral neuropathy and drugs associated with Stevens-Johnson syndrome had nine protein targets in common. Another study merged protein-protein interactions, gene-to-adverse events (AEs) associations, and knowledge of drug-protein targets to train a random forest model that identified drugs with the greatest connectivity to AEs[21]. Their analysis showed improved prediction of AEs when combining their approach, SubNet, with medication-wide association studies (MWAS) assessing genes associated with four AEs[21]. Another network based approach used a shortest-path technique for *in silico* predictions for drug repurposing[22]. A network propagation technique created drug pathways and collapsed these pathways into phenotype vectors, though, they did not use this paradigm to be predictive of drug safety and efficacy[23]. These foundational studies demonstrated that interaction networks are a rich source of pathway information that can identify molecular mechanisms for drug safety and efficacy.

Here we constructed drug pathways using protein-protein interactions, and we annotated these pathways with the phenotypes–diseases and off-target effects–associated with the pathway genes using a novel algorithm–PathFX. We demonstrated the utility of PathFX by creating pathways for marketed drugs and identified interaction paths from the drug’s target(s) to genes associated with the marketed indication of the drug. We benchmarked PathFX’s performance using a published set of marketed drugs and quantified our ability to relate a drug to its disease indication. We applied the algorithm to two tasks. First, we strengthened adverse event signals in the FDA Adverse Event Reporting System (FAERS) by searching for drug pathways containing an association to a reported adverse event. Second, we identified repurposing opportunities for marketed drugs and tested these identifications using existing off-label drug use and clinical trial data. We created a tool for better understanding drug safety and efficacy and PathFX may have the potential to aid in regulatory review and therapeutic development decisions.

## Results

### A tissue non-specific interaction network and the PathFX formalism

Recent work in identifying a drug’s marketed disease, or indication, from protein interactions found that this identification was maximized by considering protein interactions that are in close proximity to a drug’s target [22,23,24]. Thus, we hypothesized that protein-protein interactions that are proximal to a drug’s target(s) could provide insight into mechanisms of drug safety and efficacy.

To create drug interaction pathways, we pulled interaction data from iRefWeb[15], Reactome[25], PharmGKB[6], and a curated set of predicted drug-protein binding data (see Methods section). We merged and scored (explained in methods) these data to yield an interaction network of 25,604 nodes and 318,644 edges. The number of interactions and interaction score distributions are in S1 Fig.

**Fig 1:**
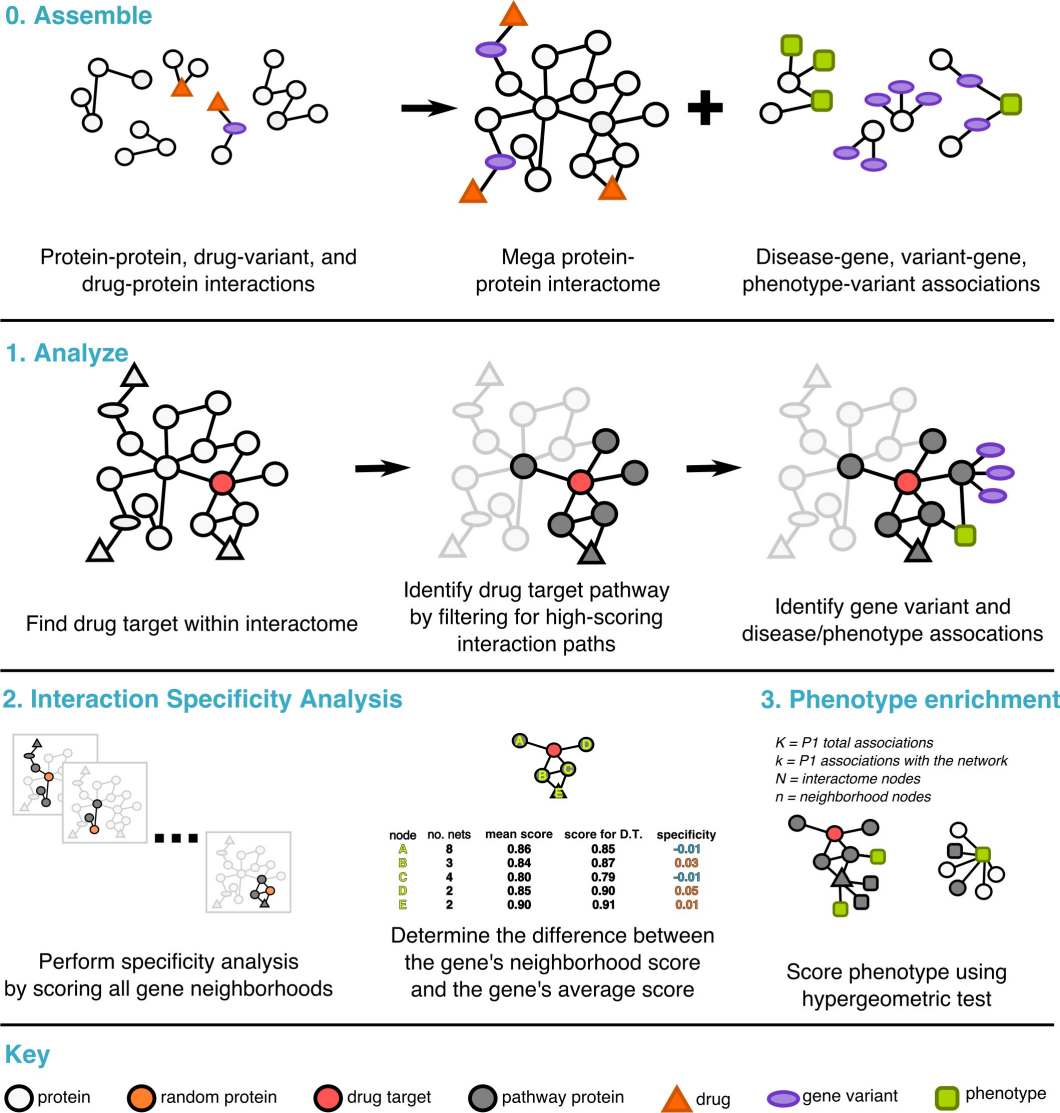
The PathFX formalism for defining drug pathways. We first assemble interaction data (top; step 0), and then applied a depth-first algorithm to define relevant protein interaction pathways (middle; step 1). We benchmarked these pathways using interaction specificity analysis (bottom, left; step 2). Lastly, we performed phenotype enrichment to explore what diseases and phenotypes existed in these local neighborhoods (bottom, right; step 3). Circles indicate proteins. Red circles are drug targets, white circles are interactome proteins, grey circles are intermediate proteins included in the drug pathway, orange triangles represent drugs, purple circles represent gene variants (not applicable in all pathways), and green rectangles represent phenotypes.

Our algorithm, PathFX, selects a drug target’s most relevant interaction edges (local interaction neighborhoods), merges neighborhood networks from all drug targets, and then identifies enriched phenotypes–which could represent either safety or efficacy phenotypes–in the interaction neighborhood (Fig 1, and usage summary in S4 Fig). In this context, safety phenotypes included associations such as adverse events or side effects (e.g. “pancreatitis”, “adverse weight gain”), and efficacy phenotypes included disease associations (e.g. “diabetes”, “major depressive disorder”); some phenotypes (e.g. “hypertension”) could belong to both of these groups. To identify which phenotypes are associated with the drug target network, we merged data from multiple sources: DisGeNet[9,10], Phenotype Genotype Integrator (PheGenI)[26], ClinVar [27], OMIM [8], and PheWas [11,12]. In this process we controlled for multiple biases as follows:

1. We reduced the inclusion of high-degree, and highly-studied hub proteins (e.g. P53, or ubiquitin) in all networks (further explained in methods). We called this process interaction specificity analysis.
2. As previous research suggested that disease modules with fewer than 25 genes were too fragmented for interactome analysis[28], we removed phenotypes associated with fewer than 25 total genes.
3. We retained phenotypes in drug pathways of interest only if the phenotype’s p-value was more significant than would be expected by chance based on the structure of our network.

PathFX uses a threshold parameter for selecting proteins included in each drug network. We derived this threshold based on the available data and did not tune the parameter to improve identification accuracy (explained in *An optimal threshold parameter for the tissue non-specific network* in Methods).

### A case study in network application: Metformin’s network is highly associated with diabetes phenotypes and is not dependent on drug target associations

**Fig 2:**
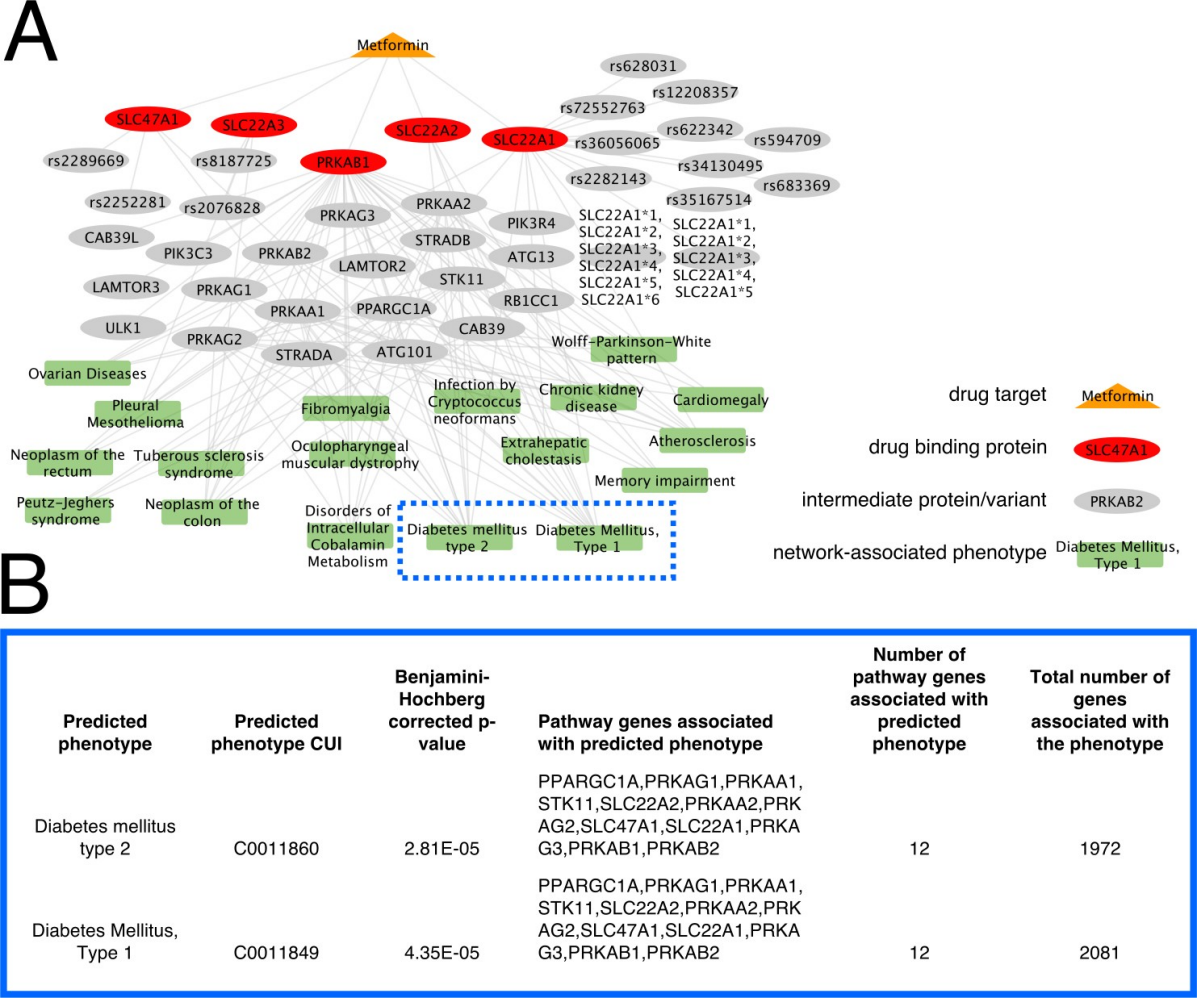
PathFX identified disease indications for Metformin. (**A**) PathFX identified a pharmacodynamic pathway for Metformin. The drug (orange triangle) is connected to protein binding targets (red circles). PathFX identified additional genes and variants (grey circles) and associated this network with phenotypes (green boxes). Edges reflect protein-protein interactions or protein-disease associations. (**B**) Selected phenotypes associated with the Metformin network highlight similarities to the drug’s marketed disease indication (all associations in S1 Table).

We first applied the PathFX method to the diabetic medication, metformin. DrugBank[29] listed five protein targets for metformin—SLC22A2, SLC22A3, PRKAB1, SLC47A1, and SLC29A4 –for metformin that were in our interactome (note that some of these are transport proteins that may be included because metformin inhibits them, not as a pharmacological effect). We created a drug interaction network pathway based on all listed proteins using PathFX. This yielded a 25-protein final neighborhood (20 proteins + 5 drug targets) significantly associated with 18 phenotypes (Fig 2A, S1 Table). Diabetes mellitus type 2 and diabetes mellitus type 1 are both associated with the metformin pathway via interactions with 12 genes (Fig 2B, S1 Table). Metformin’s protein targets were not sufficient to describe the association to diabetes mellitus type 1 and diabetes mellitus type 2 when we analyzed phenotypic associations with these targets. However, with the full 25 protein network identified by PathFX, we recovered the association to diabetes mellitus type 1 and diabetes mellitus type 2.

### A benchmarking set of approved drugs

**Fig 3:**
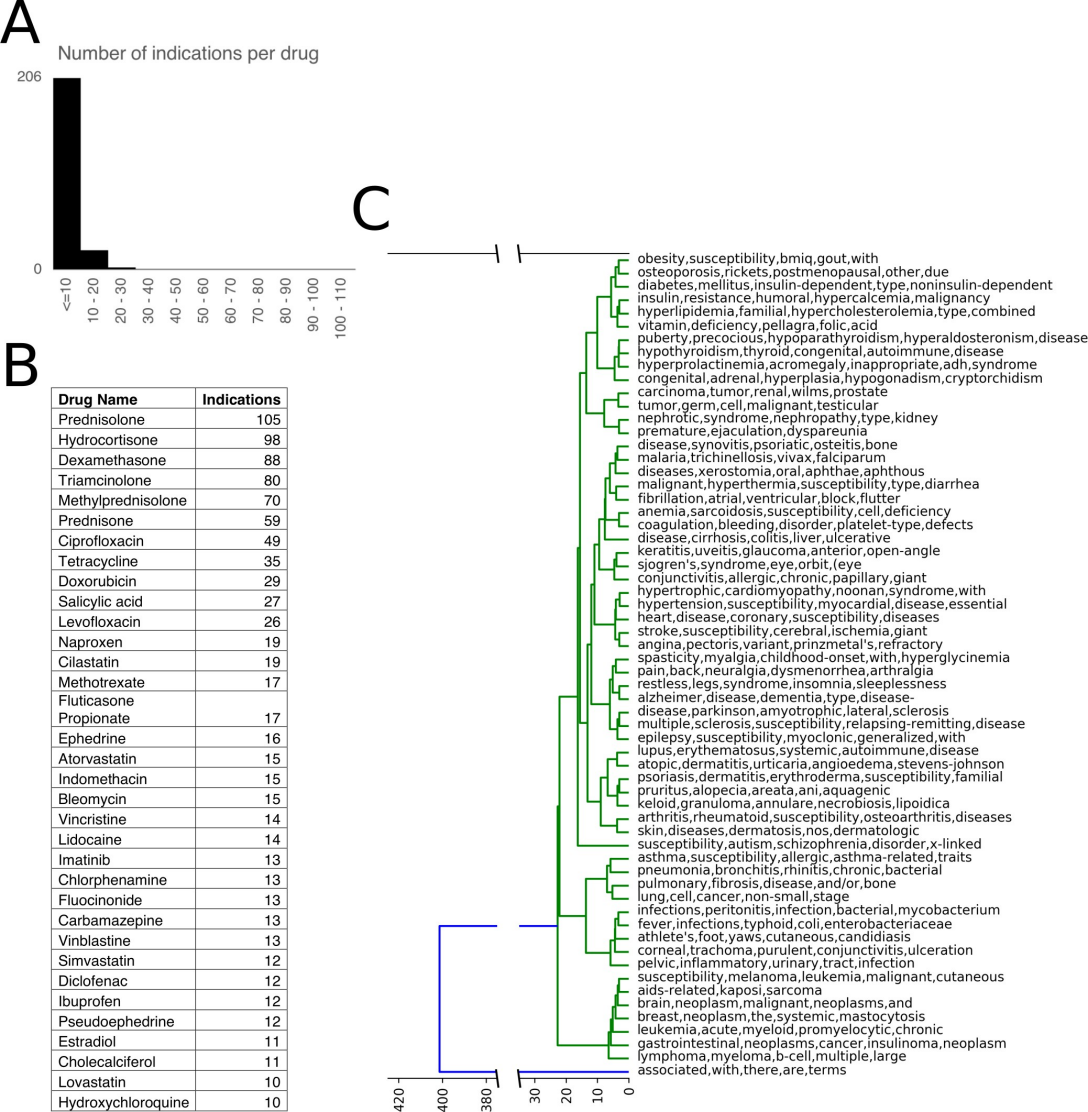
Characterization of the benchmarking drug set. Number of approved indications per drug (**A**) and top drugs by number of approved indications (**B**). A tree diagram highlighting the top disease words in each of the 62 clusters showed how the diseases were grouped (**C**).

We collected a benchmarking set of approved drugs to test our algorithm’s utility in accurately identifying diseases that the drug is known to effectively treat. This set included marketed drugs with approved disease indications. We first started with marketed, non-palliative drugs analyzed in [22] to compare our performance with this seminal work. This data set included 238 drugs associated with one or more disease indications, yielding a total of 403 drug-indication pairs. We augmented this dataset by using repoDB[30] to add additional approved indications for the original drug set (we excluded data from repoDB where the trial was terminated or ongoing); using repoDB, we added 1353 drug-indication pairs, yielding a total of 1756 drug-indication pairs for testing. The full list of drugs and approved indications are included in S1 File and consists of a list of drugs associated with one or more disease indications. Most drugs were approved for fewer than 10 indications, though, prednisolone and hydrocortisone are used to treat 105 and 98 indications respectively (S1 File, Fig 3A & 3B).

The dataset included drugs used to treat 594 indications. We binned these 594 indications into 62 clusters based on the semantic similarity of the disease indications. We used the interactive mmlite interface to metamap[31], a highly configurable program developed to map biomedical text to the UMLS Metathesaurus, to map diseases to the nearest Unified Medical Language System (UMLS) CUI (Concept Unique Identifier) identifier. We selected the UMLS terminology because this system had the greatest coverage of phenotypes in our dataset and contained mappings from many popular languages (such as MedDRA). We then clustered these diseases based on ontological, semantic similarity using the UMLS::Similarity package in Perl[32] (cluster membership in S1 File, Fig 3C). For instance, cluster four contained two CUI terms–C0497327, C0002395 –that mapped to 24 Alzheimer’s and dementia phenotypes (S1 File). Cluster five contained five CUI terms–C0042842, C0042875, C0030783, C0016412, C0936215 –that mapped to nine diseases associated with vitamin deficiency. When testing PathFX, we analyzed and reported whether the algorithm identified the drug’s original, un-clustered indication, and also reported results based on the indications’ cluster to assess trends in the types of diseases where we had better identification capacity.

### PathFX identifies disease indication for marketed drugs and improves sensitivity

**Fig 4:**
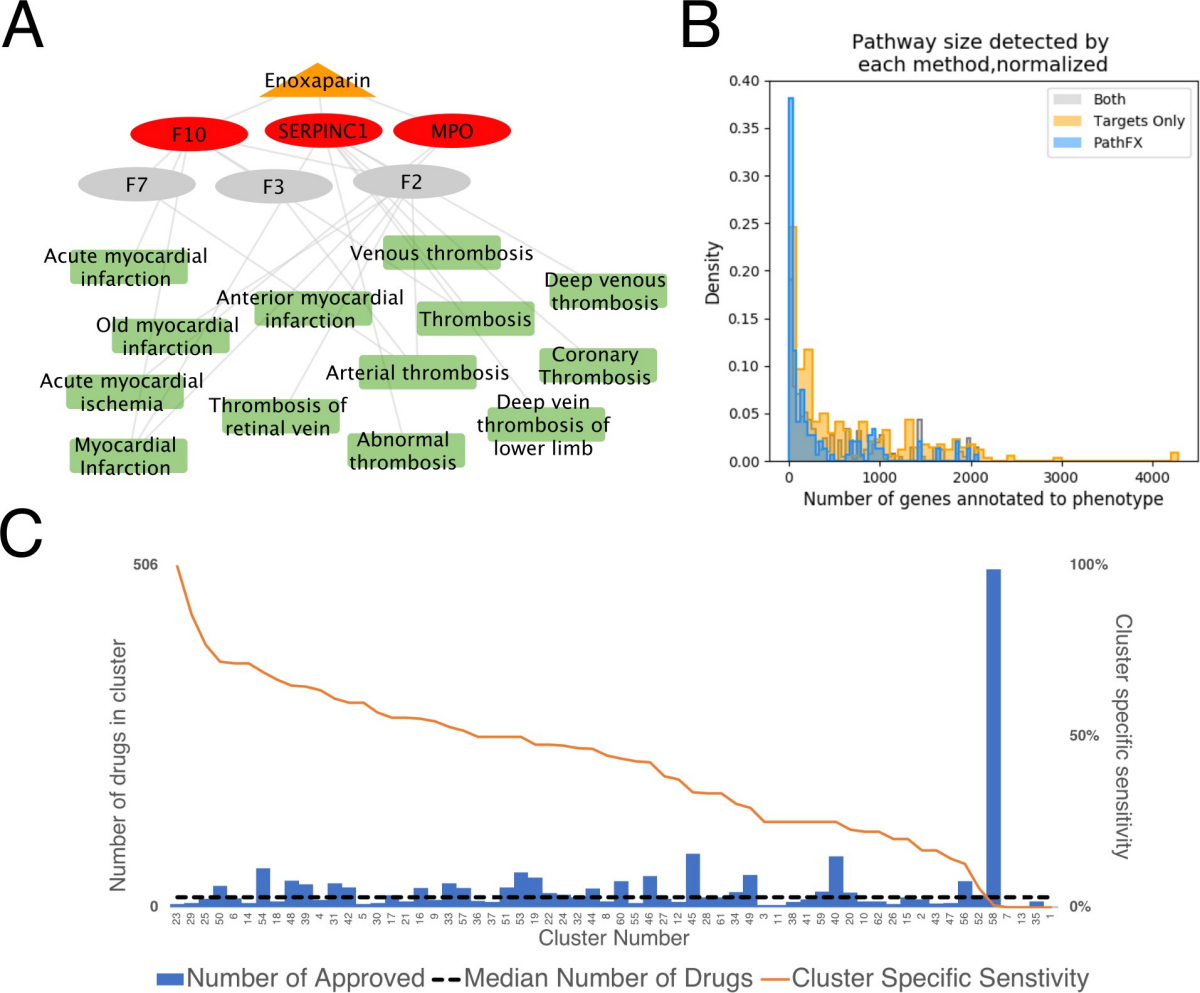
Characterization of PathFX performance. PathFX identified associations between enoxaparin and deep vein thrombosis and myocardial ischemia (**A**). Each method identified phenotypes depending on the number of genes associated with the phenotype (Kruskal-Wallis statistic 33.6, p-value = 5.04x10^-8^); PathFX identifications are skewed towards phenotypes with fewer gene associations relative to identifications by targets alone (Mann-Whitney-U statistic = 33089, p-value = 1.43x10^-8^) (**B**). PathFX identification rate per disease cluster (**C**). The orange line indicates the cluster-specific sensitivity, the bars represent total drugs in each cluster, and the dashed line represents the median number of diseases (15 diseases) in each cluster.

We used the UMLS::Similarity tools for determining if PathFX identified phenotypes that matched the drug’s marketed indication. In this case, we regarded a match as any phenotype significantly associated to the network; for most drugs, PathFX identified multiple phenotypes as statistically significantly associated to the drug’s network. For each drug, we pulled these significant phenotypes from our PathFX analysis as above, and matched these identified phenotypes to CUI identifiers, also using mmlite[31]. We measured semantic similarity using Lin distance in the UMLS::Similarity package[32]. For example, the drug enoxaparin is indicated for deep vein thrombosis and myocardial infarction; our algorithm identified that enoxaparin’s drug pathway was significantly associated with “deep venous thrombosis” (direct match, semantic similarity = 1.0), and other similar diseases such as “thrombosis” (semantic similarity = 0.8615), “venous thromboembolism” (semantic similarity = 0.6853), and “myocardial infarction” (semantic similarity = 0.9377) (full results in S1 File, Fig 4A). Of the 1756 drug-indication pairs, PathFX could not create an interaction network for two pairs (tolazamide + diabetes type 1, and tolazamide + diabetes type 2) because this drug’s target was not mapped to a gene symbol in our interactome. For 389 drug-indication pairs, metamap was unable to map the marketed indication to a CUI term so we could not assess whether the PathFX identified indications matched the marketed indications (cluster number 58 in Fig 4C and in S1 File). This left 1366 pairs for further analysis.

For this analysis, PathFX found pathway information for 171 of the 403 drug-indication pairs (42.4%) from Guney et al[22] and 558 of the 1364 (40.9%) eligible drug-disease pairs in our expanded set of benchmarking drugs; this is our best estimate of PathFX sensitivity. At the drug level, 141 out of 236 drugs (there were two of the original 238 drugs without sufficient binding information in DrugBank) had at least one identified phenotype that was similar to one of the drug’s marketed indication(s) (59.8%). Guney et al[22] reported a sensitivity of 15.4% (they matched 62 of 403 drugs-indication pairs), demonstrating improved sensitivity from our drug-target-centric approach.

For comparison with PathFX, we analyzed the disease associations of the drug targets alone without the local interaction information. Using only drug targets, we identified statistically significant associations between 751 drug and disease pairs (54.98% sensitivity). Of these pairs, 409 were also identified using PathFX pathway information, leaving 342 drug-disease pairs that were identified by targets alone and 147 drug-disease pairs that were only identified when pathway information was included from PathFX (S1 File). After merging gene-to-phenotype associations from multiple data sources, each phenotype had a set of associated genes and the size of this gene set distinguished which diseases each method detected (Kruskal-Wallis statistics = 33.6, p-value = 5.04x10^-8^) (Fig 4B). PathFX was biased towards selecting phenotypes with fewer genes (median gene set size of 90 genes); targets-only analysis was biased to selecting phenotypes with more associated genes (median gene set size of 342.5 genes) (Mann-Whitney-U statistic: 33089, p-value 1.43x10^-8^) (Fig 4B). The median gene set size where both methods detected the phenotype was 257 genes.

For completeness, we calculated positive and negative predictive values (PPV, NPV) (S2 File). To calculate PPV and NPV, we made a conservative assumption that any phenotype associated with a drug that was not a marketed disease indication was a false positive. Because PathFX was designed to search broadly for drug-associated phenotypes, the PPV and NPV were deflated and inflated respectively (S3 Fig).

We analyzed PathFX identifications in the context of the 62 disease clusters (Table 1, Fig 4C). For 58 of the 62 clusters, PathFX found pathway evidence supporting the drug’s marketed indication for at least one of the drug-disease pairs assigned to that cluster (Table 1, S1 File). For instance, the top cluster contained three CUI terms which mapped to five disease phenotypes diseases: inappropriate adh syndrome, acromegaly somatic, hyperprolactinemia, acromegaly, and prolactin excess (CUI terms C0021141, C0001206, and C0020514) (Table 1). There were five drug-indication pairs for these diseases: tolvaptan (inappropriate adh syndrome), octreotide (acromegaly somatic), bromocriptine (hyperprolactinemia), bromocriptine (acromegaly somatic), and cabergoline (hyperprolactinemia). PathFX identified phenotypes for all five drug-disease pairs. The cluster with the second highest identification rate contained four CUI terms that mapped to seven disease terms, which are pathophysiologically distinct: alcohol withdrawal delirium, restless legs syndrome, premenstrual dysphoric disorder, insomnia, nicotine dependence, late insomnia, sleeplessness. There were seven drug-indication pairs included in this cluster: gabapentin (restless legs syndrome), ropinirole (restless legs syndrome), rotigotine (restless legs syndrome), diphenhydramine (late insomnia), estradiol (premenstrual dysphoric disorder), nicotine (nicotine dependence), and diazepam (alcohol withdrawal syndrome). PathFX identified the original, un-clustered phenotype for six of the seven pairs (PathFX did not identify estradiol’s association to premenstrual dysphoric disorder) (Table 1).

**Table 1:** Example disease clusters with high cluster-sensitivity. In disease lists, ‘|’ is a delimiter to separate disease names. In drug lists, if a drug is listed multiple times, these listings reflect that the drug is intended to treat multiple indications in the specified cluster.

Cluster Number	Diseases	CUIs	Number of Approved	Approved Drug List	Number of Drugs Identified	Identified Drug List	Cluster Specific Sensitivity
23	inappropriate adh syndrome| Hyperprolactinemia, 615555 (3)| Acromegaly, somatic, 102200 (3)|Hyperprolactinemia|Acromegaly|Prolactin excess	C0021141, C0001206, C0020514	5	Tolvaptan, Octreotide, Bromocriptine, Bromocriptine, Cabergoline	5	Tolvaptan, Bromocriptine, Octreotide, Bromocriptine, Cabergoline	100.0%
29	Alcohol Withdrawal Delirium|Restless Legs Syndrome|Premenstrual Dysphoric Disorder|Insomnia|Nicotine Dependence|Late insomnia|Sleeplessness	C0035258, C0917801, C0520676, C0028043, C0001957	7	Gabapentin, Ropinirole, Rotigotine, Diphenhydramine, Estradiol, Nicotine, Diazepam	6	Gabapentin, Rotigotine, Ropinirole, Diphenhydramine, Nicotine, Diazepam	85.7%
25	Transient hypothyroidism|Goiter|Hypothyroidism, congenital, nongoitrous, 5|Hypothyroidism|{Autoimmune thyroid disease, susceptibility to, 2} (2)|{Autoimmune thyroid disease, susceptibility to, 3}, 608175 (3)|Thyroiditis|Congenital Hypothyroidism|Hypothyroidism, congenital, due to thyroid dysgenesis or hypoplasia, 218700 (3)|Hypothyroidism, Thyroidal, With Spiky Hair And Cleft Palate|{Autoimmune thyroid disease, susceptibility to, 4} (2)|Iatrogenic hypothyroidism|HYPOTHYROIDISM, CONGENITAL, DUE TO THYROID DYSGENESIS|Congenital goiter|Endocrine System Diseases|Autoimmune endocrine disease|Disorder of endocrine system|Congenital hypothyroidism|Focal thyroiditis| {Autoimmune thyroid disease, susceptibility to, 3}, 608175 (3)| Hypothyroidism, congenital nongoitrous, 5, 225250 (3)|Hypothyroidism in pregnancy|Myxedema|Other endocrine disorders|Abnormality of the thyroid gland|{Autoimmune thyroid disease, susceptibility to, 1} (2)|Goiter, multinodular 1, with or without sertoli-leydig cell tumors|Severe hypothyroidism|Thyroid Diseases	C0010308, C0014130, C0027145, C0040147, C0040128, C0020676, C0018021	13	Liothyronine, Hydrocortisone, Progesterone, Liothyronine, Triamcinolone, Hydrocortisone, Prednisone, Methylprednisolone, Prednisolone, Dexamethasone, Liothyronine, Liothyronine, Liothyronine	10	Liothyronine, Progesterone, Hydrocortisone, Liothyronine, Dexamethasone, Hydrocortisone, Prednisone, Liothyronine, Liothyronine, Liothyronine	76.9%
50	Pneumonia|Rhinitis, Vasomotor|Staphylococcal Pneumonia|Healthcare associated pneumonia|Mycoplasma pneumonia| {Allergic rhinitis, susceptibility to}, 607154 (3)|Sore Throat|Chronic bronchitis|Rhinitis|Pneumonia due to Gram negative bacteria|Common Cold|Streptococcal pneumonia|Bronchitis, Chronic|Bacterial pneumonia|Familial cold-induced inflammatory syndrome 1, 120100 (3)|Bronchitis|Sinusitis|Rhinitis, Allergic, Seasonal|Gangrenous pneumonia|Pneumonia, Bacterial|nasal scleromas|Pharyngitis|Pneumonia due to methicillin resistant Staphylococcus aureus|Chlamydial Pneumonia	C0339959, C0155862, C0032308, C0032302, C0004626, C0035468, C0035460, C0031350, C0006277, C0008677, C0009443, C0032285, C0035455, C0037199	32	Levofloxacin, Levofloxacin, Levofloxacin, Levofloxacin, Ciprofloxacin, Cilastatin, Levofloxacin, Ciprofloxacin, Ephedrine, Chlorphenamine, Promethazine, Pseudoephedrine, Diphenhydramine, Tetracycline, Ephedrine, Tetracycline, Aminophylline, Theophylline, Arformoterol, Dyphylline, Tiotropium, Epoprostenol, Ephedrine, Chlorphenamine,Isoprenaline, Salicylic acid, Pseudoephedrine, Tetracycline,Epoprostenol, Ephedrine, Pseudoephedrine, Tetracycline	23	Diphenhydramine, Pseudoephedrine, Chlorphenamine, Promethazine, Ephedrine, Tetracycline, Ephedrine, Tetracycline, Aminophylline, Theophylline, Dyphylline, Tiotropium, Pseudoephedrine, Chlorphenamine, Isoprenaline, Ephedrine, Salicylic acid, Epoprostenol, Tetracycline, Ephedrine, Pseudoephedrine, Epoprostenol, Tetracycline	71.9%
6	Allergic conjunctivitis papillary conjunctivitis, giant vernal conjunctivitides|Allergic Conjunctivitis|Chronic allergic conjunctivitis	C0009766, C0009769, C0009773	14	Ephedrine, Cromoglicic acid, Hydrocortisone, Chlorphenamine, Promethazine, Nedocromil, Cyproheptadine, Pseudoephedrine, Prednisolone, Diphenhydramine, Dexamethasone, Ketotifen, Cromoglicic acid, Cromoglicic acid	10	Diphenhydramine, Pseudoephedrine, Cyproheptadine, Chlorphenamine, Dexamethasone, Ketotifen, Nedocromil,Hydrocortisone, Promethazine, Ephedrine	71.4%

For the remaining four clusters, PathFX did not identify the drugs’ marketed indication for any of the drug-indication pairs assigned to these clusters (S1 File). These clusters are numbered 7, 13, 35, and 1. Additionally, cluster 58 contained 216 disease indications, of which 123 diseases were not mapped to a CUI term.

### Strengthening adverse event signals for designated medical events from FAERS and estimating PathFX specificity

Understanding and prioritizing drug safety signals are important regulatory concerns[33,34]. The FDA Adverse Event Reporting System (FAERS) is a repository of voluntarily submitted case reports of adverse events that occur when a patient is on a particular medication. Multiple confounding variables, including comorbidities, incomplete reports, and polypharmacy[35], make it difficult to determine when a drug is causative for an AE. This makes signal detection and triaging reports difficult. Further, when associations are not directly explained by drug targets, pathway models may provide additional justification for why a drug is associated with an AE. Anticipating and identifying drug-induced AEs are critical for novel therapeutics in development as well; and thus, further characterizing which drug targets and interacting proteins may cause AEs is an important goal of this work. Designated medical events (DMEs) are serious, significant adverse drug events of special concern to regulators. As such, DMEs, selected based on expert medical review, were evaluated in this study.

To assess the utility of PathFX for signal detection, we extracted drug-adverse event pairs from FAERs for 1906 drugs across 35 DMEs and ran PathFX on these 1906 drugs. Case reports were associated with one of the 35 DMEs if the adverse event mapped to the preferred MedDRA DME term. Close synonyms were used for some DMEs to better capture reporting (e.g., “pancreatitis” was captured by “pancreatitis” and “pancreatitis acute”). PathFX identified the adverse event phenotype for the input drugs between 0.24% - 39.57%(Table 2 and S3 File), depending on the DME. For instance, in this noisy data set, 1045 drugs were reported as having an adverse association with pancreatitis. PathFX identified that 282 of these drugs were associated with pancreatitis (26.99%). Similarly, 1150 drugs were reported to have an association with myocardial infarction and PathFX identified 391 (34.00%) of these drug-DME associations. For eight of the DME phenotypes, we found no pathways associations to the reported drugs (S3 File).

**Table 2:** Drug-DME identifications; PathFX specificity.

Designated Medical Event	Number of drugs reported with DME	Number of drugs PathFX identified	PathFX identification rate	Number PathFX identified with DME, but NOT reported	Specificity
hypertension	1261	499	39.57%	112	82.64%
myocardial infarction	1150	391	34.00%	132	82.54%
hyperlipidemia	939	288	30.67%	132	86.35%
tardive dyskinesia	878	269	30.64%	87	91.54%
renal failure	1272	379	29.80%	92	85.49%
pancreatitis	1045	282	26.99%	165	80.84%
hemorrhage	1253	271	21.63%	73	88.82%
cerebral infarction	861	159	18.47%	140	86.60%
sepsis	1130	188	16.64%	112	85.57%
pulmonary edema	1084	89	8.21%	23	97.20%
seizure	1287	101	7.85%	21	96.61%
delirium	977	73	7.47%	24	97.42%
neuropathy peripheral	1036	75	7.24%	35	95.98%
insomnia	1148	53	4.62%	6	99.21%
hepatic failure	1002	35	3.49%	10	98.89%
cardiac arrest	1211	41	3.39%	7	98.99%
thrombocytopenia	1172	35	2.99%	14	98.09%
hemolytic anemia	786	20	2.54%	10	99.11%
fracture	865	21	2.43%	18	98.27%
deep vein thrombosis	882	19	2.15%	14	98.63%
rhabdomyolysis	926	10	1.08%	8	99.18%
agranulocytosis	825	8	0.97%	2	99.81%
blindness	947	9	0.95%	10	98.96%
interstitial lung disease	770	6	0.78%	3	99.74%
cellulitis	911	7	0.77%	9	99.10%
ventricular arrhythmia	601	4	0.67%	6	99.54%
respiratory depression	819	2	0.24%	1	99.91%

We used this FAERS dataset to estimate a lower bound on the specificity of PathFX. Because FAERS contains many more drug-DME associations than are real, we treated any drugs without a reported DME association as silver-standard negatives; reasoning that if a noisy sampling of the FAERS system contained no association between the drug and the DME, that these pairs were sufficient negatives. We asked how often PathFX associated a drug with a DME when no case report existed to calculate the specificity rate for the 35 DMEs in this analysis; the rate varied from 80.84%-99.91% (Table 2, S3 File). For instance, 1261 drugs were reported to have an association with hypertension, leaving 645 of our original 1906 drugs without an association to hypertension. Of these 645 silver-standard negatives, PathFX identified an association with hypertension for 112 (17.4%) of the drugs (82.64% specificity). For cardiac arrest, 1211 drugs were reported to have an association, leaving 695 drugs without an association. Of these drugs, PathFX identified seven drugs to have an association, estimating a specificity of 98.99% (Table 2).

### PathFX identifies hypotheses for drug repurposing by identifying novel drug-disease associations

PathFX identified multiple phenotypes for each drug even if the drug only has a single approved indication. We sought support for the additional identified phenotypes from two data sets: (1) a list of off-label drug uses extracted from the electronic medical record[36] and (2) drugs currently in clinical trials. In the EMR data, we found support for six drugs applied in 11 off-label indications (Table 3, listed as ‘Jung CUI’ and ‘Jung Disease’). For instance, telmisartan is indicated for hypertension, though PathFX and the EMR dataset supported the use of this drug as an anti-diabetic; this conclusion is further supported in the literature[37]. PathFX identified that thiothixene would be broadly applicable to depressive disorders beyond the indicated use for schizophrenia. PathFX also identified that etanercept, an anti-TNF-alpha drug used in auto-immune disorders, would be applicable to colitis and this identification was supported by the Jung dataset (Table 3). PathFX identified associations for 4 drug-indication pairs supported by additional clinical trials (Table 4): sunitinib for viral infections[38], erlotinib for viral infections[38], ketoprofen for lymphoedema[39], and sirolimus for dystrophic bullosa [40].

**Table 3:** PathFX identifications supported by off-label drug use. The terms ‘Jung CUI’ and ‘Jung Disease’ are terms extracted from [36] and represent the associations between drugs and diseases found the electronic health record.

DrugBankID	Drug Name	PathFX CUI	PathFX Disease	Semantic Sim Score	Jung CUI	Jung Disease
DB00966	Telmisartan	C0011860	Diabetes mellitus type 2	0.90	C0011849	diabetes mellitus, non-insulin-dependent
		C0011849	Diabetes Mellitus, Type 1	1.00	C0011849	diabetes mellitus, non-insulin-dependent
		C0020676	Hypothyroidism	0.66	C0011849	diabetes mellitus, non-insulin-dependent
DB01043	Memantine	C0242422	Parkinsonism	0.97	C0030567	parkinson disease
		C0030567	Parkinson Disease	1.00	C0030567	parkinson disease
DB00502	Haloperidol	C0002395	Alzheimer Disease	0.66	C0003469	anxiety disorders
		C0002395	Alzheimer Disease	0.78	C0011206	delirium
		C0002395	Alzheimer Disease	0.75	C0011265	dementia
		C0002395	Alzheimer Disease	1.00	C0002395	alzheimer's disease
		C1269683	Major depressive disorder	0.73	C0041696	major depressive disorder
		C0011581	Depressive Disorder	0.68	C0003469	anxiety disorders
		C0011581	Depressive Disorder	0.71	C0002395	alzheimer's disease
		C0011581	Depressive Disorder	0.65	C0005586	bipolar disorder
		C0011581	Depressive Disorder	0.85	C0041696	major depressive disorder
		C0036341	Schizophrenia	0.65	C0003469	anxiety disorders
		C0036341	Schizophrenia	0.68	C0002395	alzheimer's disease
		C0005586	Bipolar affective disorder	1.00	C0005586	bipolar disorder
DB00624	Testosterone	C0022658	Kidney Diseases	0.75	C0019693	hiv infections
DB01623	Thiothixene	C0002395	Alzheimer Disease	0.71	C0011581	depressive disorder
		C1269683	Major depressive disorder	0.85	C0011581	depressive disorder
		C0011581	Depressive Disorder	1.00	C0011581	depressive disorder
		C0036341	Schizophrenia	0.70	C0011581	depressive disorder
		C0005586	Bipolar affective disorder	0.65	C0011581	depressive disorder
		C0497327	Dementia	0.74	C0011581	depressive disorder
DB00005	Etanercept	C0009324	Ulcerative colitis	0.90	C0010346	crohn disease

**Table 4:** PathFX identifications supported by on-going clinical trials.

Drug	DB ID	Repurposed Indication	Repurposed Indication CUI	Lin Similarity	PathFX Identification	PathFX CUI	P-value
sunitinib	DB01268	Infection; Viral (Virus Diseases) [Disease or Syndrome]	C0042769	0.8392	Symptomatic human immunodeficiency virus infection	C0019693	6.98E-05
				0.6894	Kaposi's sarcoma	C0036220	8.05E-05
				0.7142	Hepatitis C	C0019196	0.000127002
				0.656	Hepatitis	C0019159	0.000176888
				0.7366	Influenza	C0021400	0.000300686
				0.7613	Acquired Immunodeficiency Syndrome	C0001175	0.000350801
				1	Virus Diseases	C0042769	0.000359268
erlotinib	DB00530	Infection; Viral (Virus Diseases) [Disease or Syndrome]	C0042769	0.7142	Hepatitis C	C0019196	6.88E-05
				0.7366	Influenza	C0021400	8.37E-05
				0.656	Hepatitis	C0019159	9.88E-05
				1	Virus Diseases	C0042769	0.000110089
				0.8392	Symptomatic human immunodeficiency virus infection	C0019693	0.000112571
				0.7027	Herpes Simplex Infections	C0019348	0.000160023
				0.7402	Acute type B viral hepatitis	C0019163	0.000160607
ketoprofen	DB01009	LYMPHOEDEMA (Lymphedema) [Disease or Syndrome]	C0024236	0.7171	Non-Hodgkin lymphoma	C0024305	6.93E-05
				0.6666	Cutaneous T-cell lymphoma	C0079773	9.90E-05
				0.6642	Hodgkin Disease	C0019829	0.000125777
				0.6577	Granuloma	C0018188	0.000128601
				0.6563	Multiple Myeloma	C0026764	0.00013971
				0.6618	Mycosis Fungoides	C0026948	0.000139898
				0.7598	Lymphoproliferative disorder	C0024314	0.000171154
sirolimus	DB00877	Bullosa; Dystrophic Epidermolysis (Epidermolysis Bullosa Dystrophica) [Disease or Syndrome]	C0079294	0.7945	Pemphigus	C0030807	0.000248621
				0.9587	Recessive dystrophic epidermolysis bullosa	C0079474	0.000421343
				0.7925	Keloid	C0022548	0.000440175

**Fig 5:**
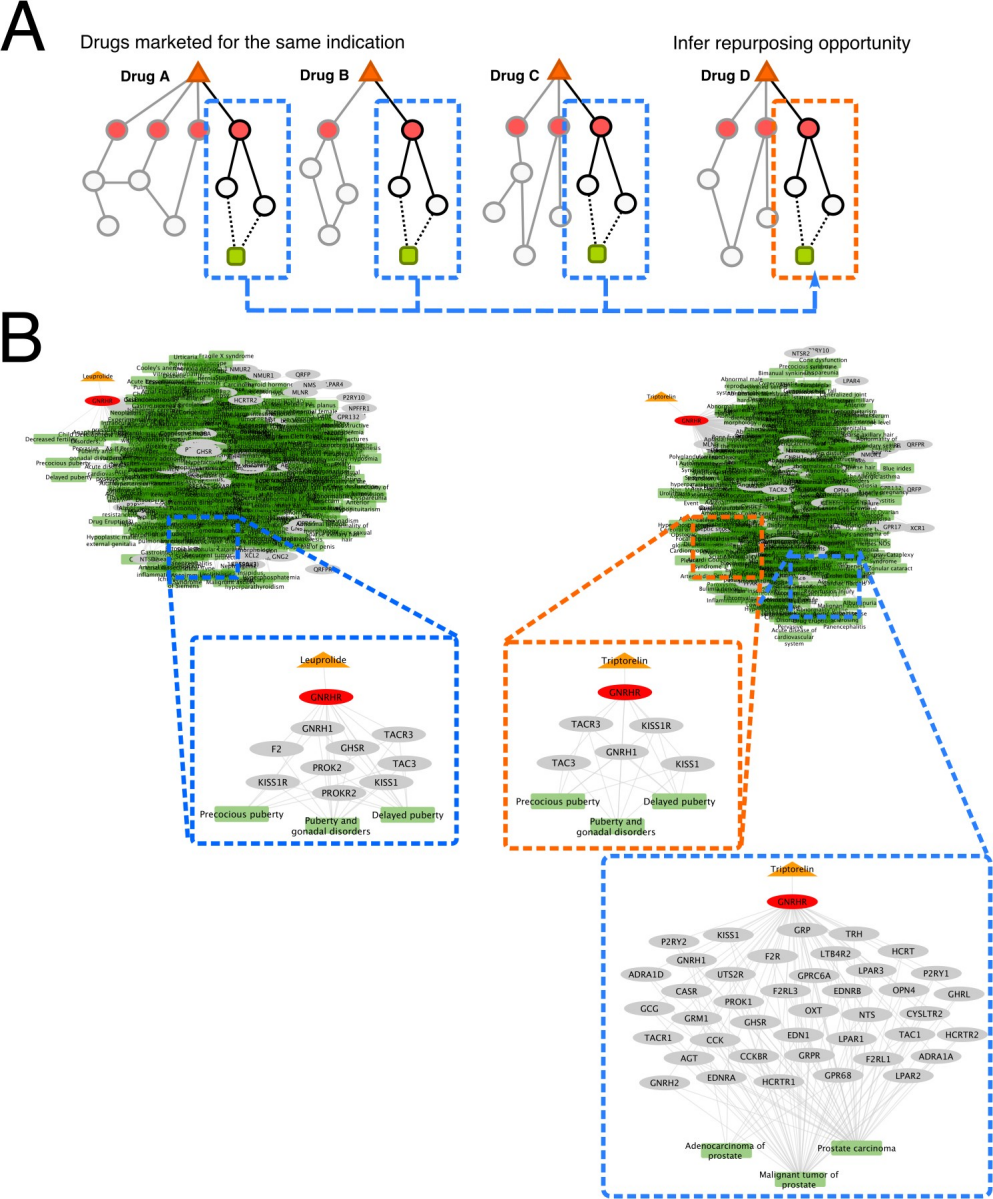
Identifying repurposing opportunities from interaction pathways. In the schematic for repurposing identifications, we identified common edges among drugs approved for a particular indication (blue outlines). We infer repurposing opportunities when a drug’s network contained the same interaction edges linking the drug target to the particular indication (**A**). An example using Leuprolide and Triptorelin: Leuprolide’s full network (top, left) and a subset of edges associated with premature puberty disorders (blue outline, middle) and Triptorelin’s full network, (top, right), and a subset of edges associated with premature puberty disorders (orange outline, middle), and prostate cancers (blue outline, bottom, left) (**B**).

Given evidence that EMR and clinical trial data supported PathFX predictions, we further scrutinized PathFX identifications to identify drug repurposing opportunities (Fig 5A); we inferred that a non-marketed indication could be a repurposing opportunity if the interaction path was found in the network of a drug marketed for this indication. We demonstrated an example with leuprolide and triptorelin, both gonadotropin-releasing hormone (GnRH) agonists (Fig 5B). PathFX also identified that the triptorelin pathway is enriched for associations with endometriosis, and these interaction pathways are supported by leuprolide’s pathway. PathFX also identified that the antispasmodic, flavoxate, could be indicated for urticaria based on interaction paths shared with cyproheptadine and promethazine, two anti-histamines already approved for urticaria. In total, we identified 2,043 new drug-disease associations for 215 drugs (S4 File). We ranked these predictions based on the number of diseases identified for a drug (top 20 in Table 5, remainder in S4 File), and the number of interaction paths supporting a drug-disease association (top 20 in Table 6, remainder in S4 File).

**Table 5:** The top 20 drugs by the number of repurposing opportunities identified by PathFX.

Drug	Number of Indications
Caffeine	63
Doxepin	63
Carvedilol	59
Halothane	54
Thiothixene	53
Haloperidol	52
Vandetanib	50
Lenvatinib	41
Cabergoline	40
Vortioxetine	37
Clomipramine	34
Tolvaptan	32
Apremilast	31
Rotigotine	31
Buspirone	29
Cabozantinib	29
Prazosin	29
Sunitinib	29
Ticagrelor	28
Gliclazide	27

**Table 6:** The top 20 drug-disease pairs based on the number of interaction paths associating the drug to the disease phenotype.

Drug	Indication	Number of Pathways Supporting
Vandetanib	Renal cell carcinoma, nonpapillary	126339
Vandetanib	Status epilepticus	82665
Tetracycline	Hypertension	64964
Ximelagatran	Arteriosclerosis	61205
Haloperidol	Parkinsonism	55008
Testosterone	Hyperlipidemia, combined, 1	42050
Ximelagatran	Asthma	39630
Haloperidol	Schizophrenia	35649
Vandetanib	Schizophrenia	26187
Diphemanil Methylsulfate	Hay fever	24435
Vandetanib	Blast Phase	21110
XL228	Blast Phase	17619
Diphemanil Methylsulfate	Bipolar affective disorder	16528
Vandetanib	Crohn Disease	16084
Vandetanib	Neuralgia	14431
Thiocoumarin	Arthritis, Rheumatoid	14408
Prazosin	Prostatic Neoplasms	14271
Tetracycline	Diabetes mellitus type 2	13934
Ximelagatran	Pulmonary Disease, Chronic Obstructive	13874
Haloperidol	Major depressive disorder	13843

## Discussion

Here we presented PathFX, a phenotypic pathways approach for characterizing drug efficacy and safety based on molecular interactions around the drug target. The algorithm characterizes the phenome around drug targets by integrating several data repositories relevant to regulatory review and therapeutic discovery. We supported our hypothesis that molecular interactions contribute to a drug’s safety and efficacy phenotypes. We successfully detected pathways that confirmed a drug’s association with its marketed disease indication and strengthened signals from adverse event reports in FAERS. In the analysis of marketed drugs, we benchmarked against a published dataset and additionally expanded this dataset to reflect updated uses of this drug set. We further discovered that PathFX identified drug phenotypes when the phenotype had relatively fewer genes associated in our data sources compared to analyzing phenotypes using the drug targets alone. We tested additional PathFX predictions using clinical trial and EMR data, and further identified novel uses for marketed drugs using networks from PathFX.

We identified associations for some phenotypes more than others. This suggested a few possibilities: incomplete data–gene-disease annotations, molecular interactions, or drug-protein binding–may prohibit the creation of pathways relating drug targets to disease associations; or marketed drugs may impact a clinically-relevant outcome to earn approval but may not be supported by genetic epidemiology studies. Incomplete knowledge of all drug-binding proteins limited our ability to construct complete drug pathways. In our database of drug-binding proteins taken from DrugBank [29], the mean and median number of proteins bound by a drug is 2.66 and 1.0 respectively. Indeed, more recent work estimates the mean and median number of proteins bound by a drug to be 329 and 38 respectively [41], suggesting that incorporation of more drug-protein interactions could improve our network predictions. Standardized drug-binding profiling could greatly improve the predictions from these algorithms. PathFX did not identify mechanism of action for all disease clusters. It is not surprising that PathFX did not identify bacterial infections, given that we are using a human protein-protein interactome. Cluster 13 contained brain cancer indications and suggested that the drug-target centric approach is not sufficient for describing efficacy for these anti-brain cancer therapies.

There are some limitations of our method: the model does not consider tissue specificity and is biased to selecting phenotypes with fewer gene annotations. Future work will consider incorporating tissue-specific interaction networks such as the GIANT networks [42] and consider screening drugs for binding across these tissues. PathFX quantifies the overlap between drug pathways and disease phenotypes but does not indicate directionality (helpful or harmful) between the drug and the pathway. Using a non-directional analysis enabled a broader discovery process given fewer directional molecular interaction networks. Compared to analyzing phenotypic associations to drug targets alone, PathFX was biased to select phenotypes with fewer genes associated and this is likely due to the statistical approach of our method: Starting with a smaller list (e.g. just the drug targets) increases the chance of finding a statistically significant association to phenotype for which there are many associations in the whole network. Conversely, starting with a larger list of proteins (e.g. using proteins from PathFX networks), decreases the chance of finding a statistically significant association to a phenotype with many proteins distributed in the interactome network. PathFX identified phenotypes where there is significant overlap with the network and where there are relatively fewer associations to the phenotype in the entire interactome network. We estimated algorithm specificity with a silver-standard data set of drugs not reported in FAERS. We recognize the limitation of this assumption because sampling biases and drug usage affect whether or not a drug is reported in FAERS in addition to our assumption that the drug does not cause an adverse event. However, we lacked sufficient gold-standard, true negatives with which to estimate specificity.

Our approach is not the first network biology tool for describing drug function, though, it does have different downstream applications. The comparator approach [22] reported lower sensitivity than PathFX. This could reinforce the role of incomplete data in creating pathways for marketed drugs or drug effects beyond the underlying disease pathway, as mentioned previously. Additionally, our improved performance could have resulted from the more permissive approach of our algorithm. The motivating question for regulatory review was “what biological evidence supports the validity of an observed adverse event?” in the post-market setting, and “what clinical trial assessments might be needed?” for vigilant detection of safety issues in the pre-market, investigational drug setting. In this paradigm, PathFX sampled disease signals around a drug target and was not constrained to find the right answer such as in the shortest-path method in Guney et al[22]. For our regulatory context, our expansive search was a positive design feature for understanding biological evidence supporting adverse events.

We demonstrated utility of PathFX in a pertinent regulatory context. Our analysis of FAERS data proved useful in detecting if and how a drug could cause a particular DME. We did not apply all available statistical filters to the FAERS data before analysis. Our intention was to apply PathFX as a biologically-motivated tool for signal detection and to develop PathFX as a complementary approach to other statistical methods; in practice, filtering using statistical approaches prior to PathFX application is a viable research strategy as well and using PathFX alone is not sufficient for identifying a causative relationship between a drug and AE. There were multiple DMEs for which we could not identify an interaction pathway. Though, for some of these diseases, further structured expansion of acceptable terms could improve prediction accuracy. For instance, the Ontology of Adverse Events (OAE)[43] provides a structured language for relating symptoms, findings, and measurements to disease and could be a useful tool for expanding the capacities of PathFX. In practice, FAERS reports do not contain research-ready disease language, and thus it is important to map and relate changes in symptoms to relevant diseases. For example, the DME, suicide attempt, would be difficult to identify with our method directly, but is likely related to depression and anxiety. Structured language relating these phenotypes would improve prediction accuracy for these phenotypes. Improved ontology incorporation is an aspiration for the project but was beyond the scope of this work.

Our findings reflect a trend in network biology to leverage drug pathways for repurposing approaches, though, the techniques are imperfect for describing all drugs. PathFX identified additional diseases beyond the marketed indications. Without a data set of true negatives (e.g. the drug was tested in condition X and did not work), it is difficult to systematically test and reject network predictions. Instead, we leveraged molecular interaction paths relating marketed drugs to their relevant disease genes and used these paths to identify possible repurposing opportunities. This prudent approach limited repurposing hypotheses to indications for which drugs already exist. Further, literature evidence supports some of the associations such as tetracycline and hypertension [44], but tetracycline induces an undesirable hypertension phenotype. We discovered these associations because we do not yet have a means for discerning directionality (e.g. a drug improves the phenotype, or a drug aggravates the phenotype). Future work will address this question.

The PathFX paradigm may be useful for both regulatory and pharmaceutical industry stakeholders to validate targets and enhance pharmacovigilance activities. We designed and tested our algorithm’s utility for one regulatory task: strengthening signals from adverse event reports in FAERS. Additionally, the drug-target-centric approach is useful for drug targets in development and may be used as a filter for identifying potential safety concerns and for confirming a sufficient association with disease. In contrast to therapeutic development through high-throughput screening, PathFX epitomizes the paradigm of identifying drug candidates based on biological rationale and supports the pathway relevance of a drug target.

## Materials and methods

### Interactome assembly, and scoring

We downloaded data from iRefWeb version 13.0 human, Reactome, and PharmGKB. We chose iRefWeb because the source contains interactions from BIND, BioGRID, CORUM, DIP, IntAct, HPRD, MINT, MPact, MPPI, and OPHID. We extracted protein-protein interactions from http://irefindex.org/wiki/index.php?title=iRefIndex, drug-variant interactions from PharmGKB, and protein-protein interactions from Reactome. We scored iRefWeb protein-protein interactions using the MIScore framework[45] and removed low-scoring interactions below the median score value, 0.244, to save memory in later computations. We kept the relative weighting of each score component equal (i.e. K_m_ = K_p_ = K_t_ = 1). The scoring framework represents the amount of evidence supporting the interaction of two proteins: the S_m_ represents the method used to detect the interaction and is higher for more dedicated experimental techniques. S_p_ reflects the number of publications supporting an interaction. This score increases with the number of publication and plateaus. S_t_ reflects the interaction type. Because we only used ‘direct’ interactions, this score is always 1.

$$S_{MI}=\frac{K_{m}S_{m}(cv)+K_{p}S_{p}(n)+K_{t}S_{t}(cv)}{K_{m}+K_{p}+K_{t}}$$

We adapted the MIScore framework for PharmGKB data and used publication, and ‘clinical evidence’ to score drug-variant relationships. Whenever an interaction with a variant was added to our network, we also added an interaction edge from the variant to the gene and scored this interaction as 0.99, the maximum possible score in the interactome. We used the following equation where *K*_*p*_ = *K*_*e*_ = 1, *S*_*p*_ was the same as published in [45]. S_e_ reflects the clinical level evidence available from PharmGKB and we crafted a scoring framework similar to [45].

$$S_{MI}=\frac{K_{p}S_{p}(n)+K_{e}S_{e}(cv)}{K_{p}+K_{e}}$$

$$S_{e}({cv}_{i})={log}_{\left(b+1\right)}(a+1)$$

$$a=\sum \left(scv_{i}\;x\;n_{i}\right)$$

$$b=a+\sum Max(Gscv_{i}))$$

Where *scv*_*’1A’*_ = 0.99, *scv*_*’1B’*_ = 0.86625, *scv*_*’2A’*_ = 0.7425, *scv*_*’2b’*_ = 0.61875, *scv*_*’3’*_ = 0.495, and *scv*_*’4’*_ = 0.2475. Because interactions in PharmGKB only receive one level of clinical evidence, a and b collapse to:

$$a=scv_{i};b=a+\sum scv_{i}$$

We adapted this scoring framework for Reactome pathways using the following equation:

$$S_{MI}=\frac{K_{m}S_{m}(cv)+K_{p}S_{p}(n)+K_{t}S_{t}(cv)}{K_{m}+K_{p}+K_{t}}$$

*S*_*p*_ was the kept the same as in [45]. We derived a scheme for the method component, *S*_*m*_, where *scv*_*’direct_complex’*_ = 1.00, *scv*_*’neighboring_reaction’*_ = 0.66, *scv*_*’indirect_complex’*_ = 0.8, *scv*_*’reaction’*_ = 1.0, and *scv*_*’unknown’*_ = 0.05.

$$S_{t}({cv}_{i})={log}_{\left(b+1\right)}(a+1)$$

$$a=\sum \left(scv_{i}\;x\;n_{i}\right)$$

$$b=a+\sum Max(Gscv_{i}))$$

Because the two maximum scoring categories were ‘direct_complex’ and ‘reaction’, the Max(Gscv_i_) term = 2.0. Because Reactome interactions did not include a method of detection but are curated interactions, we gave these interactions a *cv*_*i*_ = 0.8. Because they all received the same ‘method’ score, calculating *a* and *b* yields a *S*_*m*_ = 0.615.

We lastly incorporated predicted drug to protein binding data based on PocketFEATURE [46] where drug-protein pairs were scored based on the similarity between the drug’s known targets and other protein targets from the Protein Data Bank[47] (See methods below). PocketFEATURE has been extensively validated on predicting drug protein interactions in multiple applications [46,48,49]. In all cases, we estimated interaction scores based on the quality of evidence available; these edge scores were fixed before applying PathFX and we did not alter these parameters to improve prediction accuracy.

### Phenotype, disease, and variant data

We downloaded variant and phenotype association data from PheWAS[11,12], disease to gene associations from DisGeNet[10,11], Phenotype-Genotype Integrator (PheGenI) [26], ClinVar[27], and OMIM[8], and eQTL data from the GWAS catalogue[7]. We collapsed all phenotype names to CUI identifiers using MetaMap lite and took the union of all data sources to create our source of gene to phenotype annotations. This yielded a database associating 29785 genes to 20524 phenotypes.

To look for enriched phenotypes, we used a Fisher’s exact test and Benjamini-Hochberg multiple hypothesis correction to assess whether a disease or phenotype had more associations to the network genes relative to the total number of associations in the interactome. We filtered out disease or phenotypes associated with fewer than 25 genes. Recent work suggested that current interactomes are insufficient for analyzing disease pathways with fewer than 25 genes[28]. More specifically, interactomes are incomplete and these missing interactions reduce the ability to find pathways between smaller disease modules. This recent estimate discovered that missing interactions disproportionally affect phenotype pathways with fewer than 25 genes and thus, we eliminated these phenotypes knowing that our interaction network was insufficient for studying these phenotypes.

We further filtered phenotypic predictions by deriving a p-value threshold from networks created with randomly-selected, druggable proteins. We realized that if any random set of input drug-targeting proteins could discover a statistically-significant association to a given phenotype, that associating a real set of drug proteins with this phenotype would reflect bias in our data instead of a biologically-meaningful result. To assess this bias in our data, we created 100 networks using randomly selected drug targets from the intersection of all drug-targeting proteins in DrugBank and ran PathFX using these targets as inputs (i.e. using the same statistical approaches to assess phenotypes that are significantly associated to networks created with random inputs). Because the distribution of p-values from the 100 randomizations was not normally distributed, we used the median value as the threshold p-value for determining if a drug network was associated to a phenotype. The number of randomly selected input proteins matched the number of targets of the drug of interest. PathFX retained a phenotype if the association is more significant than the p-value threshold for that phenotype run with the same number of random, input protein targets.

When analyzing drug targets alone, we again used the Fisher’s exact text, the Benjamini-Hochberg correction, and filtering relative to the expected p-value threshold.

### Depth-first network search

We created a depth-first search tool which ‘walked’ away from a drug’s protein target through the interactome and evaluated these paths using the multiplicative sum of all interaction edges between a gene and the target. Genes with a path score above the threshold were retained in the drug pathway. We expedited the search with “fast-tracking”. This process reflects the fact that molecules exist in highly interconnected pathways and assumed that we could reduce the searchable interaction spaces by looking for molecular redundancies. As the search explored an interaction path, fast-tracking searched the remaining que of interaction paths for genes that had already been added to the network and added these interaction paths to the network. Interaction edge scores were used from the scoring system above. We used specificity analysis to determine an optimal threshold for the interactome (S2 Fig, explained below). In the case of multiple protein targets, we created a pathway around each protein target, and merged these neighborhoods to create the full protein network.

### Interaction specificity analysis

We evaluated the interactome specificity by comparing a gene’s path score to all possible path scores for that gene. To measure all path scores, we created pathways for all genes in the interactome network, treating each molecular entity as a drug target and creating a pathway as described above. We used these empirically-derived scores to calculate an enrichment score for an entity in the pathway of a real drug target by subtracting the average path score to that gene from the gene’s score in the drug pathway (Fig 1, ‘Interaction Specificity Analysis’).

### An optimal threshold parameter for the tissue non-specific network

We selected an optimal threshold by evaluating gene specificity at threshold values from 0.7 to 0.9. At each of these values we created a drug pathway around the drug’s protein target(s), calculated the gene specificity, and then tabulated the fraction of genes that are specific to a drug target (i.e. have a specificity score > 0). We plot the normalized histograms of specificity values in S2A Fig and a distribution of the proportion of specific paths at each threshold value in S2B Fig.

### PathFX code

The PathFX code is available at: https://github.com/jenwilson521/PathFX. Using the algorithm requires minimal inputs and creates a network and several association files as depicted in S4 Fig. The user provides three inputs: 1. an analysis name. 2.the name of the drug. 3. an optional list of proteins (if the drug-binding proteins are not in DrugBank or the user wishes to complete a more specific analysis).The algorithm creates a set of output files: 1. networks for individual target proteins and a merged interaction network combining networks from each target proteins. These files are tab-delimited files with one interaction per line and the score for that interaction. 2. An association table containing one significantly-associated network phenotype, a p-value for that association, and network genes associated with that phenotype. 3. A table listing the database source for individual phenotype-gene associations.

### Mapping to Common Unique Identifier (CUI) terms

For all diseases, these phenotypes were mapped to CUI terms using Metamap lite[31]. This was the same process used in assembling the phenotype dataset.

### Semantic similarity and disease clustering

We downloaded the UMLS Metathesaurus, version 2017AA and used the Perl packages UMLS::Interface[32] and UMLS::Similarity[32] to measure the lin distance between diseases in a set. For the gold-standard drug set, we calculated a matrix of similarity values for all approved indications and we used SciPy in Python to perform hierarchical clustering. We identified 62 as the optimal number of clusters using the elbow method. For visualization of the dendrogram, we counted the top five disease-associated words in each cluster. To determine how well PathFX identified a drug’s approved indication, we again used the umls-similarity.pl scripts to calculate similarity between the approved indication and the PathFX identified phenotypes.

### Positive and Negative predictive values

Because the number of true positives and true negatives varied for each drug and for each phenotype, we calculated the PPV and NPV separately for each drug and for each phenotype. To calculate PPV, we assumed that false positives were any PathFX identified phenotype that was not a marketed indication. The PPV was the ratio of correctly identified marketed indications to the total of marketed indications and additional PathFX phenotypes. To calculate NPV, we considered any phenotype from our dataset that was not a marketed disease indication to be a true negative. The NPV was the ratio of these true negatives to the sum of the true negatives and the unidentified, marketed indications. When calculating PPV for each phenotype, we assumed that a false positive was any drug identified by PathFX to be associated to the phenotype but was not marketed for that phenotype. When calculating NPV for each phenotype, we considered as true negatives any drugs not marketed for or identified by PathFX to be associated with the phenotype.

### Statistical comparison of performance between PathFX and drug targets alone

We calculated the number of genes associated with the original marketed indication for drug-disease pairs identified by PathFX only, targets only, or identified by both methods. We first used a Kruskal-Wallis test implemented in the Python package, SciPy, to determine that these populations were not from the same distribution. We tested the hypothesis that the targets-only analysis was biased towards diseases with more genes using the Mann-Whitney-U statistic implemented in the Python package, SciPy.

### FAERS drug-DME pair data extraction

The FDA Adverse Event Reporting System (FAERS) data was extracted using the Oracle Health Sciences Empirica Signal software. Although, the software is not publicly available, we used only publicly-available data at the time of analysis (2004-2017Q3). All drugs that had at least one case reported for the 35 MedDRA Preferred Terms identified as Designated Medical Events by review and medical experts at the FDA were included in this analysis.

### Repurposing drugs for marketed indications

From the successfully matched drug-indications pairs, we created a catalogue of interaction paths that supported the association between these drugs and their approved indications. We searched through remaining drug pathways and asked if any associations with the drugs’ non-marketed indications were supported by the catalogue of interaction paths. These non-marketed associations became our cohort of repurposing predictions.

### Predicting drug protein interactions

The Drug-binding Dataset collects 984 high-quality 3D structures (x-ray resolution higher than 2.5 Å) that co-crystalized with FDA approved small molecule drugs (non-nutraceuticals), representing binding environments of 284 distinct drugs [50]. The Human Off-target Dataset comprises 2271 proteins representing a non-redundant representative set (90% percent identity) of human proteins and their close homologs that have high quality 3D structures (x-ray resolution higher than 2.5 Å) in PDB[51]. We have applied PocketFEATURE[46] to predict the probability of binding between the 284 drugs and the 2271 potential off-targets. PocketFEATURE uses the FEATURE representation to calculate site similarities by aligning microenvironments between two sites. A more negative score suggests binding site similarity and thus a higher probability of drug binding to a site similar (off-target) to its known binding site. Given a pair of drug and off-target, we used an average score of similarity between the binding sites and the off-target. For each drug, we generated a profile of its binding probability to each of the 2271 potential off-targets.

## Supporting information

S1 File Summary of PathFX associations for marketed drugs. The file contains the starting list of drug-disease associations from marketed drugs, associations between drugs and diseases discovered by PathFX, a clustered summary where drug-disease associations are binned based on the semantic similarity of the diseases, a comparison to associations found from using drug targets without network information, and a listing of disease associations discovered by PathFX, targets-only, or both methods and the number of genes annotated to these diseases.
(XLSX)

S2 File Positive and negative predictive value calculated by drug and by disease CUI term. (XLSX)

S3 File Analysis of FAERs case reports using PathFX. A summary of where PathFX associated a drug to a reported adverse event and a summary of false positives from the silver-standard set of negative cases (i.e. drugs without a reported association to an adverse event in FAERs).
(XLSX)

S4 File Summary of repurposing predictions. A summary of drug-disease associations ranked by the number of pathways supporting an association and by the number of predicted alternative indications.
(XLSX)

S1 Fig A tissue-non-specific interactome for identifying drug pathways. The table shows the number of interactions obtained from each database (**A**) and the distribution of edge scores (**B**). These edge scores represent the probability of an interaction given the available evidence.
(TIF)

S2 Fig Generalized interaction specificity analysis results. We created all drug target paths using the depth-first search at threshold values ranging from 0.7–0.9. We calculated gene specificity relative to all paths created for that gene and plot specificity values as normalized histograms (**A**). The total number of paths and the percent enriched (scored >0) are indicated in each figure legend. We additionally plot the fraction of specific paths against the total number of paths (**B**). Shading is a linear gradient corresponding to the threshold value (0.9 = dark purple, 0.7 = light purple). A threshold of 0.77 was used for all further analyses.
(TIF)

S3 Fig Density histograms of positive and negative predictive values for targets only analysis (top row, gray) and PathFX analysis (bottom row, blue).
(TIF)

S4 Fig Summarized schematic for PathFX usage. The user provides three inputs: 1. an analysis name. 2. the name of the drug. 3. an optional list of proteins (if the drug-binding proteins are not in DrugBank or the user wishes to complete a more specific analysis). The algorithm outputs a set of output files: 1. networks for individual target proteins and a merged interaction network combining networks from each target proteins. These files are tab-delimited files with one interaction per line and the score for that interaction. 2. An association table containing one significantly-associated network phenotype, a p-value for that association. 3. A table listing the database source for individual phenotype-gene associations.
(TIF)

S1 Table PathFX identified phenotypes for Metformin. A list of phenotypes significantly associated with the Metformin network.
(PDF)
