# Supplementary figures and images for: PathFX provides mechanistic insights into drug efficacy and safety for regulatory review and therapeutic development
Source: PLoS Comput Biol. 2018 Dec 7;14(12):e1006614. doi: 10.1371/journal.pcbi.1006614 (PMC6285459; doi:10.1371/journal.pcbi.1006614)

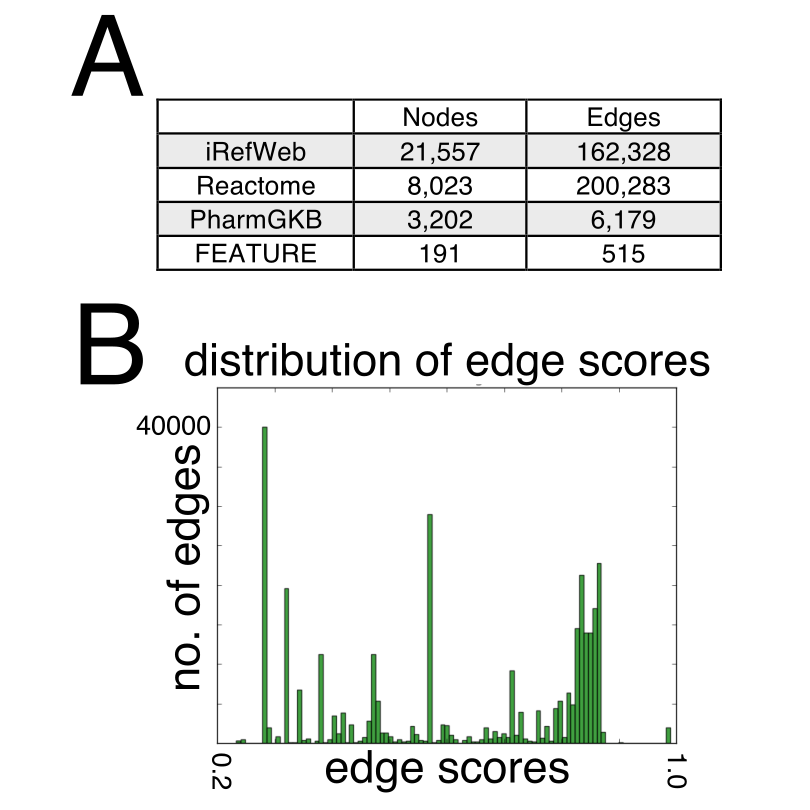

Supplement: S1 Fig — The table shows the number of interactions obtained from each database (A) and the distribution of edge scores (B). These edge scores represent the probability of an interaction given the available evidence. (TIF) [file pcbi.1006614.s005.tif]

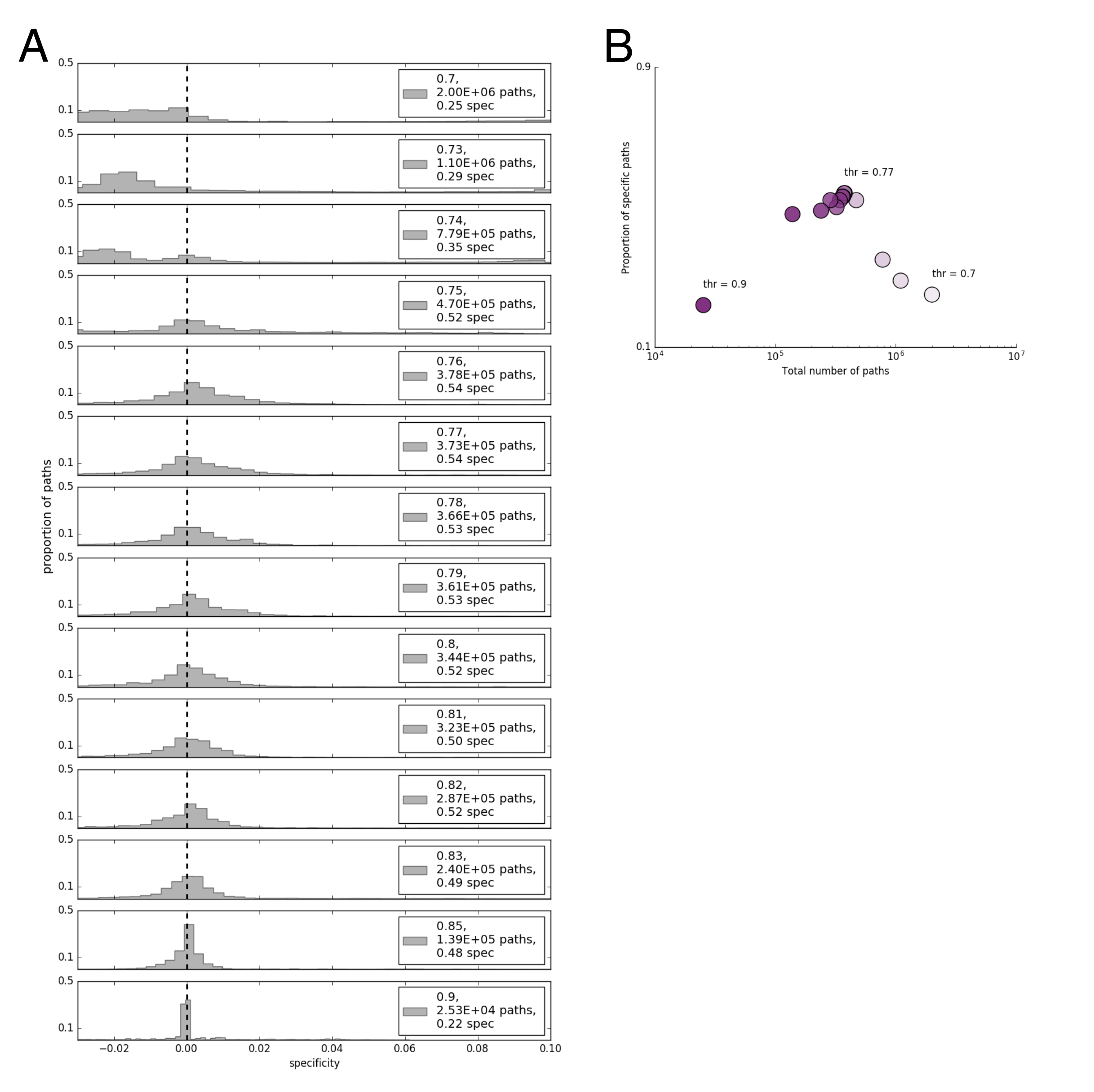

Supplement: S2 Fig — We created all drug target paths using the depth-first search at threshold values ranging from 0.7–0.9. We calculated gene specificity relative to all paths created for that gene and plot specificity values as normalized histograms (A). The total number of paths and the percent enriched (scored >0) are indicated in each figure legend. We additionally plot the fraction of specific paths against the total number of paths (B). Shading is a linear gradient corresponding to the threshold value (0.9 = dark purple, 0.7 = light purple). A threshold of 0.77 was used for all further analyses. (TIF) [file pcbi.1006614.s006.tif]

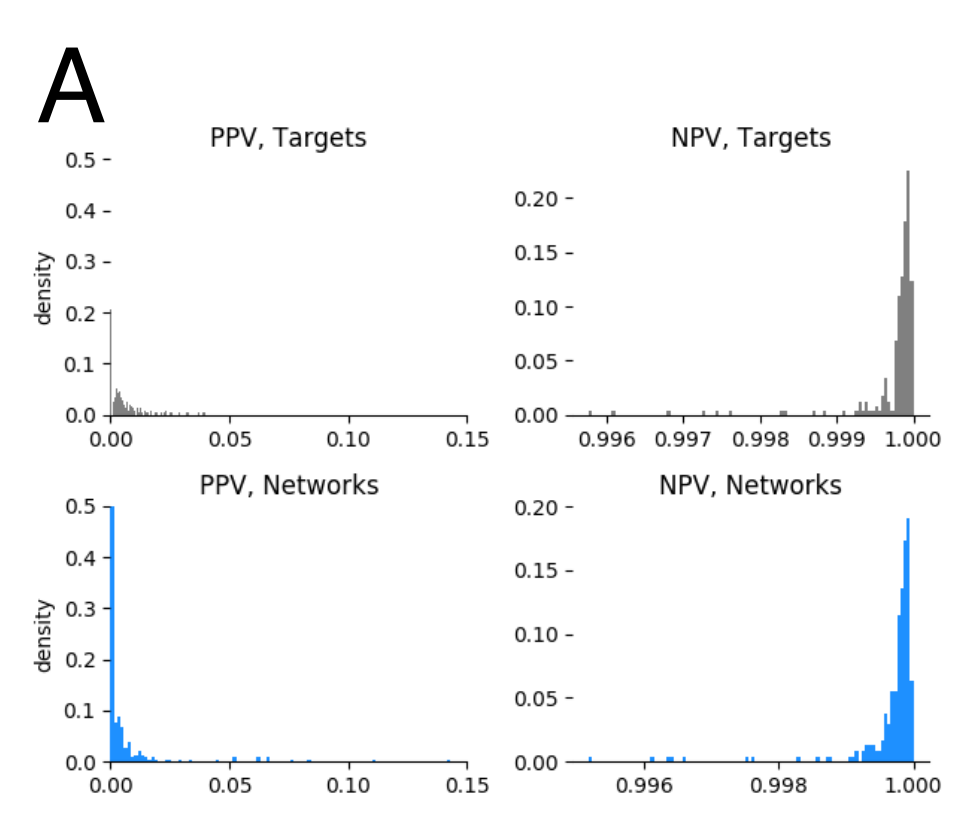

Supplement: S3 Fig — Density histograms of positive and negative predictive values for targets only analysis (top row, gray) and PathFX analysis (bottom row, blue). (TIF) [file pcbi.1006614.s007.tif]

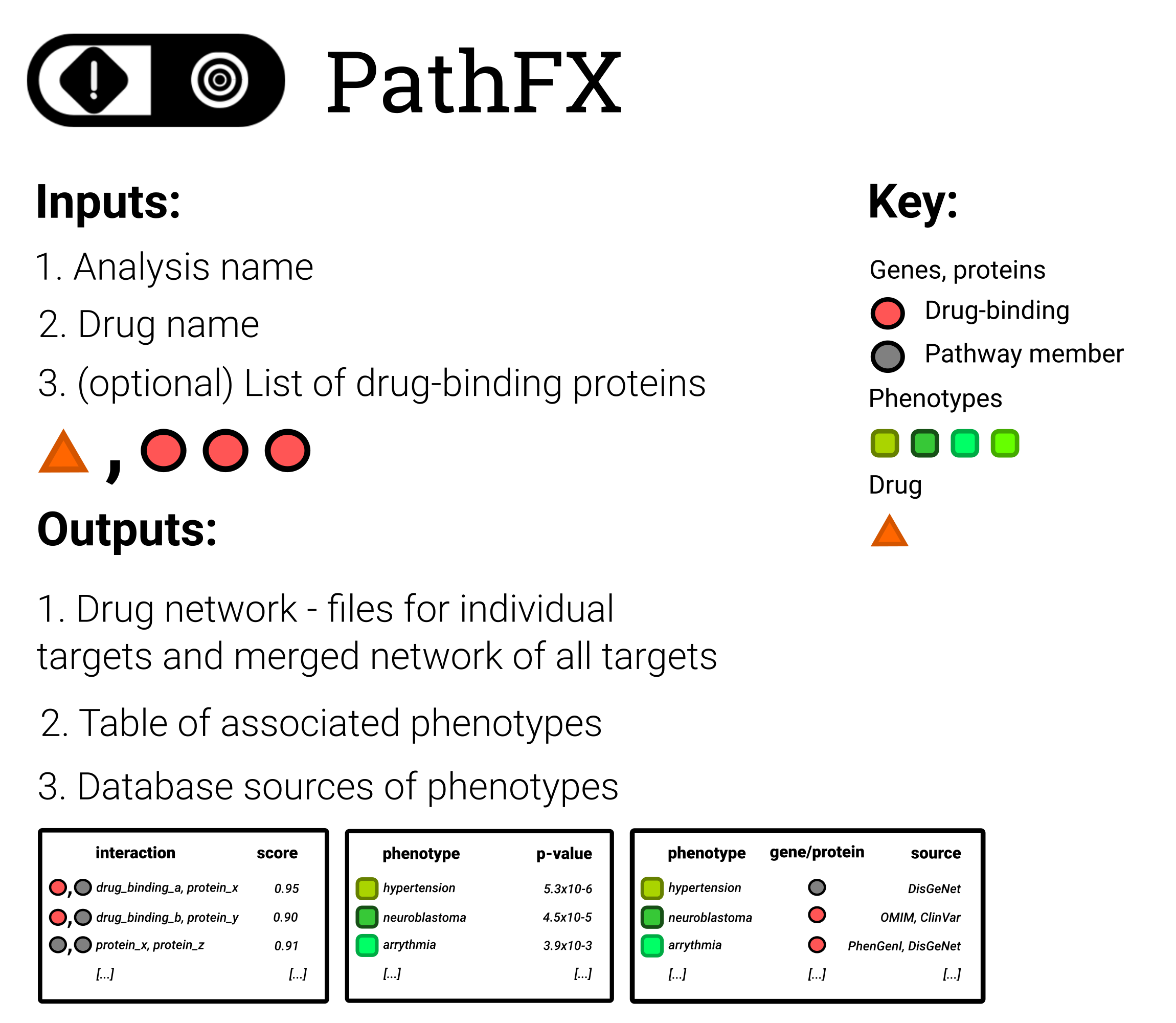

Supplement: S4 Fig — The user provides three inputs: 1. an analysis name. 2. the name of the drug. 3. an optional list of proteins (if the drug-binding proteins are not in DrugBank or the user wishes to complete a more specific analysis). The algorithm outputs a set of output files: 1. networks for individual target proteins and a merged interaction network combining networks from each target proteins. These files are tab-delimited files with one interaction per line and the score for that interaction. 2. An association table containing one significantly-associated network phenotype, a p-value for that association. 3. A table listing the database source for individual phenotype-gene associations. (TIF) [file pcbi.1006614.s008.tif]
