# Supplementary material for: PathFX provides mechanistic insights into drug efficacy and safety for regulatory review and therapeutic development
Source: PLoS Comput Biol. 2018 Dec 7;14(12):e1006614. doi: 10.1371/journal.pcbi.1006614 (PMC6285459; doi:10.1371/journal.pcbi.1006614)
Supplement: S1 Table — A list of phenotypes significantly associated with the Metformin network. (PDF) [file pcbi.1006614.s009.pdf]

Supplementary Tables

Identified phenotype	Identified phenotype CUI	Benjamini-Hochberg corrected p-value	Pathway genes associated with identified phenotype	Number of pathway genes associated with identified phenotype	Total number of genes associated with the phenotype
Wolff-Parkinson-White pattern	C0043202	1.02E-05	PRKAA1,PRKAA2,PRKAG2,PRKAB1	4	35
Oculopharyngeal muscular dystrophy	C0270952	1.28E-05	PRKAA1,PRKAA2,PRKAB1	3	25
Fibromyalgia	C0016053	1.41E-05	PRKAA1,PRKAA2,PRKAB1	3	48
Ovarian Diseases	C0029928	1.53E-05	PPARGC1A,STK11,PRKAA2,SLC22A1,PRKAG3	5	236
Infection by Cryptococcus neoformans	C0010414	1.66E-05	PRKAA1,PRKAA2,PRKAB1	3	57
Disorders of Intracellular Cobalamin Metabolism	C0025517	2.56E-05	PPARGC1A,PRKAA1,STK11,PRKAA2,PRKAG2,PRKAB1	6	394
Peutz-Jeghers syndrome	C0031269	2.69E-05	STK11,CAB39,STRADA	3	73
Diabetes mellitus type 2	C0011860	2.81E-05	PPARGC1A,PRKAG1,PRKAA1,STK11,SLC22A2,PRKAA2,PRKAG2,SLC47A1,SLC22A1,PRKAG3,PRKAB1,PRKAB2	12	1972
Diabetes Mellitus, Type 1	C0011849	4.35E-05	PPARGC1A,PRKAG1,PRKAA1,STK11,SLC22A2,PRKAA2,PRKAG2,SLC47A1,SLC22A1,PRKAG3,PRKAB1,PRKAB2	12	2081
Tuberous sclerosis syndrome	C0041341	4.48E-05	PRKAA1,STK11,PRKAA2,PRKAB1	4	192
Extrahepatic cholestasis	C0005398	5.24E-05	SLC22A2,SLC22A1	2	30
Chronic kidney disease	C1561643	5.37E-05	PRKAA1,SLC22A2,PRKAA2,PRKAG2,PRKAB1	5	342
Atherosclerosis	C0004153	5.50E-05	PPARGC1A,PRKAG1,PRKAA1,PRKAA2,PRKAG2,SLC22A1,PRKAG3,PRKAB1,PRKAB2	9	1271
Neoplasm of the rectum	C0034885	5.63E-05	PRKAA1,STK11,PRKAG2	3	105
Neoplasm of the colon	C0009375	6.91E-05	SLC22A3,PRKAA1,STK11,PRKAG2,SLC22A1,RB1CC1,PRKAB1	7	817

Pleural Mesothelioma	C0812413	7.03E-05	SLC22A3,PRKAA1,PRKAA2,PRKAB1	4	240
Memory impairment	C0233794	7.67E-05	PPARGC1A,PRKAA1,PRKAA2,PRKAB1	4	255
Cardiomegaly	C0018800	8.57E-05	PPARGC1A,STK11,PRKAA2,PRKAG2	4	279
